# Optimizing DNA extraction protocols for bryophytes: Insights from Orthotrichaceae

**DOI:** 10.1002/aps3.70028

**Published:** 2025-11-04

**Authors:** Pablo Aguado‐Ramsay, Francisco Lara, Macarena Cuerdo, Isabel Draper

**Affiliations:** ^1^ Departamento de Biología, Facultad de Ciencias Universidad Autónoma de Madrid C. Darwin 2 Madrid 28049 Spain; ^2^ Centro de Investigación en Biodiversidad y Cambio Global Universidad Autónoma de Madrid C. Darwin 2 Madrid 28049 Spain

**Keywords:** β‐mercaptoethanol, CTAB, genomics, high‐molecular‐weight DNA, mosses, polyvinylpyrrolidone (PVP)

## Abstract

**Premise:**

Extracting high‐quality DNA from challenging plant tissues can be hindered by high levels of phenolics, carbohydrates, and other compounds that bind to or coprecipitate with DNA. Bryophytes, due to their small size, tendency to intermix, biochemical richness, and capacity to absorb environmental pollutants and heavy metals, pose unique challenges that further complicate proper DNA isolation.

**Methods and Results:**

We compared cetyltrimethylammonium bromide (CTAB)‐based extraction protocols and evaluated step‐by‐step modifications aimed at maximizing DNA yield and purity for Orthotrichaceae, one of the most diverse families of bryophytes. The final protocol was also evaluated on representatives of other bryophyte families as a preliminary exploration of its broader applicability.

**Conclusions:**

We present an optimized, rapid, and efficient DNA extraction protocol that yields high‐quality DNA suitable for high‐throughput sequencing and molecular analyses. The presented extraction protocol is likely to work well for other bryophyte taxa and may be of value for molecular analyses involving recalcitrant samples.

Advances in high‐throughput sequencing (HTS), including both whole‐genome sequencing and reduced‐representation methods such as restriction site–associated DNA sequencing (RADseq), genotyping‐by‐sequencing (GBS), or hybrid enrichment with genome skimming (Hyb‐Seq), are opening up new opportunities in the plant sciences (McKain et al., 2018; Lozano‐Fernandez, 2022; Bartoš et al., 2023; Pezzini et al., 2023). These technologies require DNA of high quality and purity, for which numerous isolation protocols are available (e.g., Doyle and Doyle, 1987; Sharma et al., 2000; Mogg and Bond, 2003; Tsubota et al., 2009). However, extracting DNA from plants often poses significant challenges due to the presence of secondary metabolites and other compounds that vary across plant groups, which affect its isolation and inhibit its proper amplification and sequencing (Jobes et al., 1995; Porebski et al., 1997; Csaikl et al., 1998; Khanuja et al., 1999; Pirttilä et al., 2001; Michiels et al., 2003; Dehestani and Kazemi Tabar, 2007; Ribeiro and Lovato, 2007; Särkinen et al., 2012; Healey et al., 2014; Aboul‐Maaty and Oraby, 2019). Although universal DNA extraction protocols have been proposed (Whitlock et al., 2008; Wang et al., 2011), a one‐size‐fits‐all approach is unlikely (Loomis, 1974). Instead, protocols are typically tailored to specific plant groups to account for their chemotypic variability. Consequently, there is a prevailing trend toward modifying existing protocols, particularly the cetyltrimethylammonium bromide (CTAB) method (Schenk et al., 2023). This is especially true for plants that have historically been less studied (e.g., Xie et al., 2023; Hossen et al., 2025).

Bryophytes are non‐vascular plants comprising about 20,000 species (Brinda and Atwood, 2025). They are rich in chemicals, some of which are not found in vascular plant species (Xie and Lou, 2009; Klavina, 2015, 2021; Sabovljević et al., 2016; Chen et al., 2018; Martínez‐Abaigar and Núñez‐Olivera, 2021; Bandyopadhyay and Dey, 2022; Dziwak et al., 2022), and can bioaccumulate many substances (Tessier and Boisvert, 1999; Elvira et al., 2020; García‐Seoane et al., 2023; Chaos et al., 2024). These traits, along with the challenge of obtaining sufficient tissue due to their small size and the tendency of species to grow intermixed, likely contribute to the frequent difficulties reported in DNA isolation (Dhyani et al., 2025). As a result, it is necessary to develop protocols specifically adapted to these organisms (Schlink and Reski, 2002; Fernandez et al., 2006; Pandey et al., 2019; Saługa, 2020). Orthotrichaceae, the third most diverse family among bryophytes, comprises around 800 species of mosses (Brinda and Atwood, 2025), most of which are epiphytes. Recent studies show that this family has a complex evolutionary history (Draper et al., 2021, 2022; Aguado‐Ramsay et al., 2022) and that there may be greater diversity than previously expected (Vigalondo et al., 2019; Lara et al., 2020; Matanov et al., 2024). Molecular phylogenetic reconstructions based on Sanger sequencing have sometimes lacked sufficient resolution, underscoring the need for HTS techniques (Aguado‐Ramsay et al., 2024; Lara et al., 2024; Matanov et al., 2025). However, we have encountered difficulties in meeting the DNA requirements of the sequencing centers, both in terms of quantity and purity. In this scenario, there is a pressing need to refine DNA extraction protocols for this group of mosses.

Although specific DNA extraction methods for bryophytes have been proposed (e.g., Werner et al., 2002; Pedersen et al., 2006), they are not widely used. The most commonly adopted approaches include commercial DNA extraction kits and modified versions of the CTAB method (Stenøien, 1999; Schlink and Reski, 2002; Fernandez et al., 2006; Mittmann et al., 2007; Soni and Kumar, 2009; Saługa, 2020). In this context, it is important to highlight the CTAB protocol proposed by Pandey et al. (2019), which is described as an adaptation of Khanuja et al. (1999) specifically designed for bryophytes. Pandey et al. (2019) evaluated different concentrations of CTAB, polyvinylpyrrolidone (PVP), and β‐mercaptoethanol (BME), and compared their protocol to several others, including the original protocol of Khanuja et al. (1999), two additional modified CTAB protocols described for bryophytes (Scott, 1999; Soni and Kumar, 2009), and a commercial kit (HiPurA Plant Genomic DNA isolation and purification kit; HiMedia, Thane, Maharashtra, India). However, while this and some additional studies have evaluated different DNA extraction protocols for bryophyte species (e.g., Schlink and Reski, 2002; Mikulášková et al., 2012; Saługa, 2020), none have focused on Orthotrichaceae. Moreover, the substantial chemical variability among plant groups complicates the extrapolation of results from such studies to this family.

Therefore, our objective is to develop an efficient DNA extraction protocol specifically tailored to Orthotrichaceae. To this end, we start from an initial reference modified CTAB protocol (hereafter referred to as the IR protocol) based on an extensive review and evaluation of existing protocols. We will compare the IR protocol with three alternatives: the Qiagen DNeasy Plant Mini Kit (Qiagen, Venlo, the Netherlands), the modified CTAB method from Pandey et al. (2019), and a hybrid protocol combining the IR protocol with optimized reagent concentrations from Pandey et al. (2019). In a first phase, we will determine which of these four protocols is best suited to our target species. In a second phase, we will further optimize the selected protocol by systematically evaluating key parameters, including different forms of PVP; varying concentrations of PVP, BME, and CTAB; as well as incubation and precipitation times, precipitation temperatures, and the addition of salts to enhance DNA precipitation. Finally, as an exploratory objective, we will test the selected protocol with a representation of other bryophyte families (Appendix S1) to gain preliminary insight into its potential applicability.

## METHODS AND RESULTS

### Sample collection and pretreatment

*Lewinskya rupestris* (Schleich. ex Schwägr.) F. Lara, Garilleti & Goffinet was selected as the model species for the following reasons: (1) it is a widespread and common species that is easy to identify, (2) it generally forms monospecific cushions, and (3) its large size facilitates the collection of ample material (Lara and Garilleti, 2014). To maximize homogeneity of variables and comparability of results, all samples were collected from a single population a few days prior to extraction. Plant tissue (20 mg) was selected from the dry apical parts of several contiguous gametophores with confirmed taxonomic identification. To eliminate potential contaminants, the samples were placed in an Eppendorf tube with 1.5 mL of distilled water, shaken in a Retsch Mixer Mill MM400 (Retsch, Haan, Germany) at 10 Hz for 1 min, transferred to a new 2‐mL round‐bottom Eppendorf tube, and left to air‐dry overnight. As powdering the samples is one of the critical steps, several strategies were tested (results not shown, but discussed below). We considered: (1) whether to grind samples wet or dry, (2) whether to freeze the samples prior to grinding, (3) the method of freezing, and (4) whether to use a mortar or an automated grinder. Based on these tests, the selected approach involved flash freezing the dry samples in liquid nitrogen, followed by pulverization in a Retsch Mixer Mill MM400 using 12 glass beads (3 mm) at 30 Hz. The grinding process consisted of two 2‐min repetitions, reversing the tube orientation between cycles.

### Initial reference modified CTAB protocol

We developed a modified reference CTAB protocol (IR protocol) for 2‐mL tubes based on an extensive review and evaluation of existing methods (e.g., Doyle and Doyle, 1987; Cullings, 1992; Healey et al., 2014; Anderson et al., 2018; Inglis et al., 2018; Aboul‐Maaty and Oraby, 2019), with particular emphasis on those previously used in HTS studies on bryophytes, especially Breinholt et al. (2021).

### First step: CTAB extraction protocol comparison

We compared the IR protocol with three isolation approaches (Table 1): the Qiagen Plant Mini Kit (following the manufacturer's protocol), the modified CTAB method from Pandey et al. (2019), and a hybrid protocol that follows the IR protocol but adjusts the concentrations of CTAB, BME, and PVP as specified by Pandey et al. (2019). Each protocol was tested on 24 replicates.

**Table 1:** Comparison of the methods and results (mean ± SD) of the CTAB‐based protocols discussed in this paper.

Phase	Step	Pandey et al. (2019)	IR protocol	Hybrid protocol
**Lysis**	**Lysis buffer**	1 mL of buffer (they use 1.5 mL per 0.5 g of sample, volume is reduced to 1 mL), 25 mM EDTA (pH 8.0), 100 mM Tris‐Cl (pH 8.0), 1.5 M NaCl, 3% CTAB, 1% BME, and 2% PVP‐40	900 µL of buffer, 20 mM EDTA (pH 8.0), 100 mM Tris‐Cl (pH 8.0), 1.4 M NaCl, 2% CTAB, 0.4% BME, and 2.5% PVP‐40	900 µL of buffer, 20 mM EDTA (pH 8.0), 100 mM Tris‐Cl (pH 8.0), 1.4 M NaCl, 3% CTAB, 1% BME, and 2% PVP‐40
	**Incubation**	60°C 1 h	55°C 1 h	55°C 1 h
**Isolation**	**CIA wash**	1 mL of CIA (24:1). Centrifuge at 10,000 rpm and 18°C for 10 min.	800 µL of CIA 24:1. Centrifuge at 14,000 rpm and 18°C for 10 min.	800 µL of CIA 24:1. Centrifuge at 14,000 rpm and 18°C for 10 min.
	**RNA treatment**	(Carried out later)	2 μL of RNase (10 mg/mL), incubate at 37°C for 30 min	2 μL of RNase, incubate at 37°C for 30 min
	**CIA wash**	Repeat wash.	Repeat wash with 700 µL of CIA.	Repeat wash with 700 µL of CIA.
	**Precipitation**	750 µL of NaCl (5 M) and 0.6 volume of chilled isopropanol and incubate for 1 h at −20°C. Centrifuge for 25 min at 4°C and 13,000 rpm. Discard supernatant.	600 µL of ice‐cold isopropanol and incubate at −20°C overnight. Centrifuge for 10 min at 4°C and 12,000 rpm. Discard supernatant.	600 µL of ice‐cold isopropanol and incubate at −20°C overnight. Centrifuge for 10 min at 4°C and 12,000 rpm. Discard supernatant.
**Cleaning**	**Pellet wash**	100 µL of 80% ethanol and centrifuge at 8000 rpm for 10 min. Discard ethanol and air‐dry pellets.	500 µL of ice‐cold 70% ethanol and centrifuge for 5 min at 12,000 rpm. Discard ethanol.	500 µL of ice‐cold 70% ethanol and centrifuge for 5 min at 12,000 rpm. Discard ethanol.
	**RNA treatment**	Dissolve pellet in 0.5 mL or high salt TE buffer (1 M NaCl, 10 mM Tris‐Cl, and 1 mM EDTA). Add 5 µL of RNase and incubate at 37°C for 30 min.	(Previously performed)	(Previously performed)
	**CIA wash**	Repeat CIA with 0.5 mL.	—	—
	**Second precipitation**	Transfer the aqueous layer and add 1 mL of cold ethanol. Centrifuge at 10,000 rpm for 10 min at 20°C. Discard supernatant.	—	—
	**Pellet wash**	100 µL of 80% ethanol, air‐dry pellets.	Repeat ethanol wash with 500 µL of 95% ethanol, air‐dry pellets.	Repeat ethanol wash with 500 µL of 95% ethanol, air‐dry pellets.
**Elution**		Add 50 µL of TE buffer.	Add 100 µL of preheated TE buffer and place in a 50°C heated block to help the pellet dissolve.	Add 100 µL of preheated TE buffer and place in a 50°C heated block to help the pellet dissolve.
**RESULTS**	**DNA yield**	777 ± 472 ng	5483 ± 1809 ng	4194 ± 1225 ng
	**A**_**260**_ **/A**_**280**_	1.97 ± 0.08	1.83 ± 0.04	1.88 ± 0.03
	**A**_**260**_ **/A**_**230**_	2.12 ± 0.41	1.66 ± 0.09	1.76 ± 0.08

Abbreviations: BME = β‐mercaptoethanol; CIA = chloroform:isoamyl alcohol; CTAB = cetyltrimethylammonium bromide; IR protocol = initial reference modified CTAB protocol; PVP = polyvinylpyrrolidone; TE = Tris‐EDTA.

**Figure 1:**
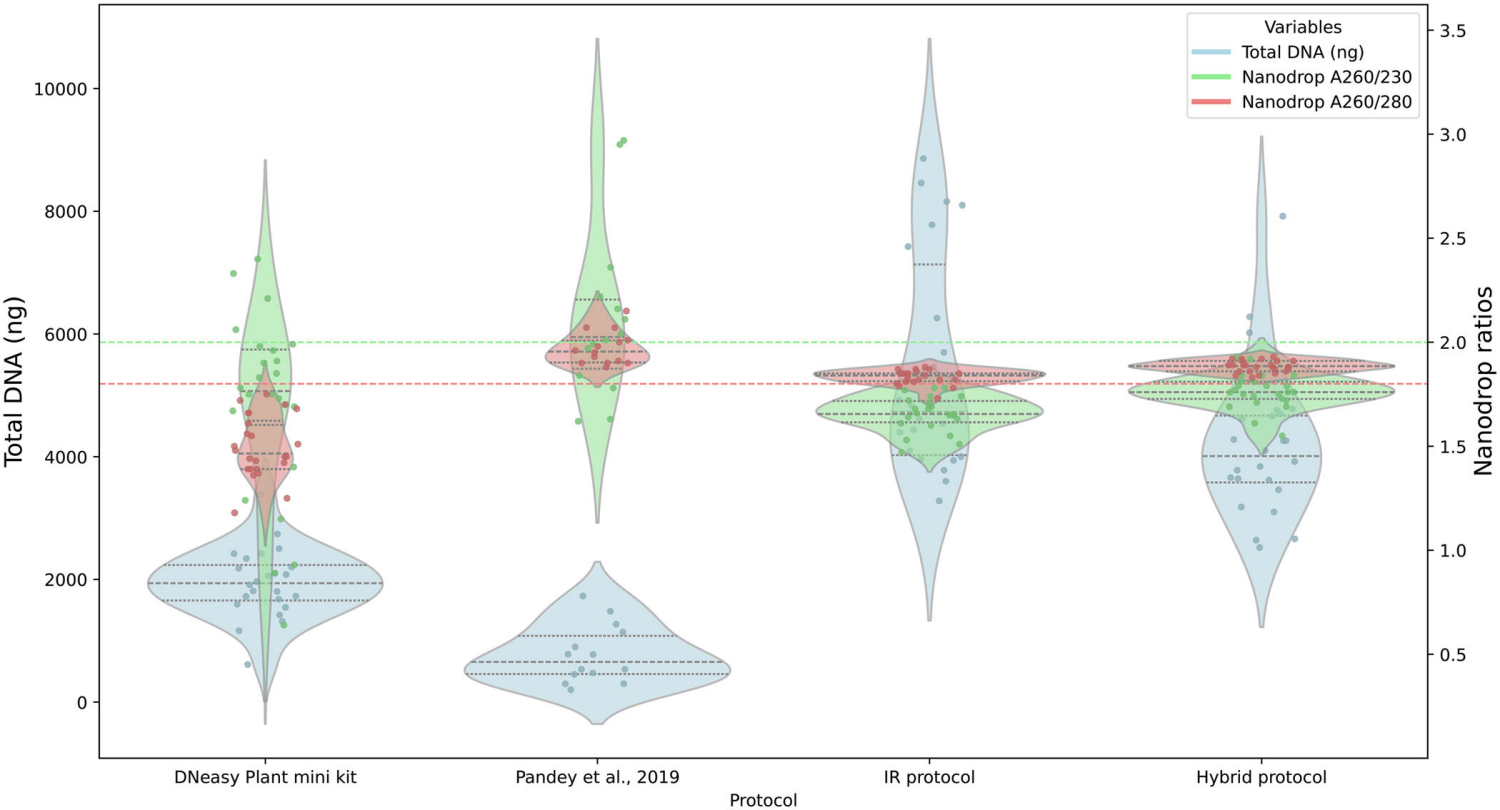
Violin plot showing results from the comparison of different DNA extraction protocols. Each point represents a value for DNA quantity or purity. Blue indicates DNA quantity, with its associated scale on the left *y*‐axis. Green and red represent DNA quality measured through the values of the A_260_/A_230_ and A_260_/A_280_ ratios, respectively, with their scales on the right *y*‐axis. The dashed green and red lines indicate the cutoff values for high‐purity measurements.

In terms of DNA yield, the IR protocol produced the highest values, followed by the hybrid protocol, the commercial kit, and finally the protocol from Pandey et al. (2019) (Figure 1, Table 1). For the ratio of absorbance at 260 nm and 280 nm (A_260_/A_280_), Pandey et al. (2019), the IR protocol, and the hybrid protocol all fell within the acceptable range, whereas the commercial kit had a much lower ratio. The highest A_260_/A_230_ ratios were obtained from the Pandey et al. (2019) protocol, followed by the hybrid protocol, the commercial kit, and the IR protocol. Most differences in DNA yield and purity were statistically significant (Appendix S2). Considering the results, we selected the IR protocol for further optimizations, as it yielded the highest amount of DNA and its A_260_/A_280_ ratio was optimal, although it showed an A_260_/A_230_ ratio below the optimal range.

### Second step: Protocol optimization

To optimize the IR protocol, we evaluated different protocol modifications using our model species (*Lewinskya rupestris*). From each experiment, we selected the best parameter to apply from that point onward. When selecting the final conditions to be applied in the optimized protocol, we sought a balance between DNA yield and NanoDrop purity ratios—placing greater emphasis on the A_260_/A_230_ purity ratio. Protocol simplicity was also considered, as it is important that the protocol can be completed within a single working day and not be excessively time‐consuming. We also acknowledge that not all laboratories have access to the same resources.

We first evaluated the use of different forms of PVP by applying three different lysis buffers containing either PVP‐10 (molecular weight [MW] 10,000 or K12), PVP‐40 (MW 40,000 or K30), or polyvinylpolypyrrolidone (PVPP). We evaluated different incubation times (1 h, 2 h, 4 h, and overnight at 55°C) and then assessed different lysis buffer concentrations (PVP‐10 was tested at 2%, 2.5%, 3%, and 3.5%; BME at 0.4%, 0.8%, 1.2%, and 1.6%; and CTAB at 2%, 2.5%, 3%, and 3.5%). For the precipitation step, we evaluated the application of sodium acetate at 0.3 M. As the salt might coprecipitate, we also evaluated the effect of including an extra 70% ethanol wash (both with and without salt). We then evaluated precipitation times (1 h, 6 h, overnight [~16 h], and two nights [~40 h]) at a standard temperature of −20°C. Based on the results obtained from these precipitation times, we also evaluated shorter precipitation periods (1.5 h, 3 h, 4.5 h, and 6 h). Finally, we evaluated different precipitation temperatures (room temperature, 4°C, −20°C, and −80°C) at a standard precipitation time of 3 h. Except for the different forms of PVP, which was done with eight replicates, all modifications were tested on six replicates.

Across all the modifications evaluated for the target model species *Lewinskya rupestris*, high DNA yield values were obtained, with an overall average of 5605 ± 1497 ng. All A_260_/A_280_ values fell within acceptable ranges, with an overall average of 1.88 ± 0.04, and in general, A_260_/A_230_ values did not exceed the threshold of 2, but had an overall average of 1.94 ± 0.08, which is acceptable. The most significant deviation was observed with the two‐night precipitation time, which exhibited a mean of 1.76 ± 0.08. Descriptive statistics and parametric and nonparametric tests for the protocol optimizations are presented in Appendix S3.

**Figure 2:**
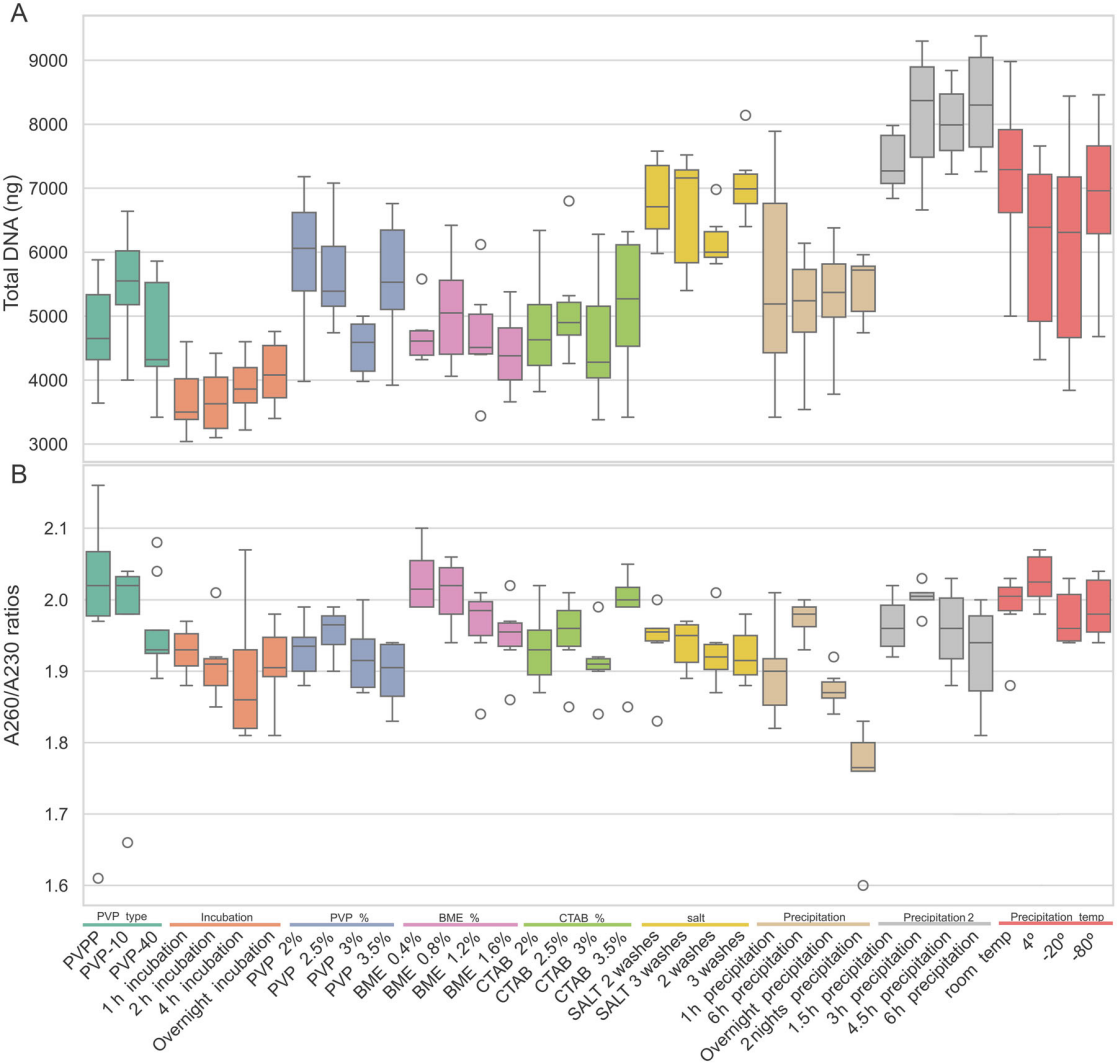
Box plot showing the results obtained for DNA yield (A) and the A_260_/A_230_ ratios (B) for the comparison of the different DNA extraction protocol modifications.

When examining the specific results of each evaluated modification (Table 2, Figure 2, Appendix S4), PVP‐10 yielded the highest amount of DNA, with all DNA showing similar purity values. Regarding incubation times, longer times seemed to result in slightly higher DNA yields while maintaining similar purity levels. We opted for a 1‐h incubation primarily to streamline the protocol, keeping it within a single working day, but also because this duration resulted in slightly higher purity. PVP concentration at 2.5% resulted in slightly higher purity with similar DNA yields. Higher concentrations of BME seemed to result in lower purities, with ANOVA and Tukey's range test results marginally significant. For this reason, we selected the initial BME concentration at 0.4%, which is also the most cost‐effective. Increasing CTAB concentrations did not demonstrate a linear relation to DNA yield and purity; we therefore selected a concentration of 2.5%, as it resulted in slightly higher DNA quantity and purity. Precipitation with salt (sodium acetate) did not seem to result in higher DNA yields, although it increases purity at a statistically significant level according to both parametric and nonparametric tests at the A_260_/A_280_ ratio. However, purity as measured by the A_260_/A_280_ ratio is within optimal ranges for all salt treatments; therefore, as its addition does not appear to be necessary, we chose the simplest and most cost‐effective option. Notably, including an extra 70% ethanol wash did not reduce DNA yield. In reference to precipitation times, a 1‐h precipitation yielded high DNA quantities, but with great variability. Although there seems to be a tendency for DNA yield to increase with longer precipitation times, A_260_/A_230_ ratios strongly and significantly decreased with longer precipitation times according to both parametric and nonparametric tests. We therefore decided to evaluate shorter precipitation times and selected a 3‐h precipitation, as it resulted in the highest DNA yields and purities. Finally, our results did not show a linear relation between precipitation temperature and DNA yields.

**Table 2:** Summary of tested steps in the CTAB protocol optimization.

Step tested	Conditions compared	Final choice	Rationale
PVP type	PVP‐10, PVP‐40, PVPP	PVP‐10	Yielded the highest amount of DNA, all showing similar purity values.
Incubation times	1 h, 2 h, 4 h, overnight	1 h	The shortest incubation time was selected to simplify the protocol, although longer periods may slightly increase DNA yield.
PVP concentration	2%, 2.5%, 3%, 3.5%	2.5%	Resulted in slightly higher purity with similar DNA yields.
BME concentration	0.4%, 0.8%, 1.2%, 1.6%	0.4%	Resulted in higher purity with similar DNA yields.
CTAB concentration	2%, 2.5%, 3%, 3.5%	2.5%	No clear trend observed. 2.5% was chosen as it yielded high DNA concentration and purity, and matches the selected PVP concentration, simplifying the protocol.
Salt addition and ethanol washes	Two or three washes, with or without salt	Two washes without salt	Although salt increased A_260_/A_280_ ratios, values remained within optimal ranges without it. Thus, the simpler option was selected.
Precipitation time	1 h, 1.5 h, 3 h, 6 h, overnight, 2 nights	3 h	There is a tendency for DNA yield to increase over time, but A_260_/A_230_ ratios strongly decreased with longer precipitation times. We selected a 3‐h precipitation, as it resulted in the highest DNA yields and purities.
Precipitation temperature	Room temperature, 4°C, −20°C, −80°C	4°C	No clear trend was observed. Precipitation at 4°C yielded slightly higher purity values and was therefore selected, although other temperatures may also be suitable.

Abbreviations: BME = β‐mercaptoethanol; CTAB = cetyltrimethylammonium bromide; PVP = polyvinylpyrrolidone; PVPP = polyvinylpolypyrrolidone.

### Final protocol evaluation

The efficiency of the optimized protocol was evaluated on several species within the family Orthotrichaceae: *Cardotiella secunda* (Müll. Hal.) Vitt, *Macrocoma tenuis* (Hook. & Grev.) Vitt, *Macromitrium archeri* Mitt., *Nyholmiella obtusifolia* (Brid.) Holmen & E. Warncke, *Orthotrichum anomalum* Hedw., *Pulvigera lyellii* (Hook. & Taylor) Plášek, Sawicki & Ochyra, *Schlotheimia ferruginea* (Burch. ex Hook. & Grev.) Brid., *Ulota coarctata* (P. Beauv.) Hammar, *U. hutchinsiae* (Sm.) Hammar, and *Zygodon hookeri* Hampe. Additionally, several other species were selected to test whether the selected protocol might work as well for other mosses: *Aulacomnium palustre* (Hedw.) Schwägr., *Bartramia pomiformis* Hedw., *Dendrocryphaea lamyana* (Mont.) P. Rao, *Dicranum scoparium* Hedw., *Polytrichum juniperinum* Hedw., and *Racomitrium aciculare* (Hedw.) Brid. We also included six replicates of *Lewinskya rupestris* for comparison purposes. The information associated with the samples can be found in Appendix S1.

Acceptable DNA yields were obtained for all the species studied (Table 3). The lowest yield was 1400 ng for *Macromitrium archeri*, while the highest was 9060 ng for *Orthotrichum anomalum*. All species had appropriate NanoDrop A_260_/A_280_ ratios, demonstrating acceptable purity levels. For the NanoDrop A_260_/A_230_ ratio, some values were slightly lower than ideal but fell in an acceptable range, with the lowest being 1.75 for *Schlotheimia ferruginea*. Electrophoresis of both extractions and PCR products showed positive results. The sequencing and nucleotide BLAST search of the *rps4* (see below, under DNA Purity and Quantity Evaluation) confirmed high‐quality data, with the exception of *M. archeri*, for which purity was not perfect, although the BLAST results still verified its identity.

**Table 3:** Efficiency test of the optimized protocol.

Species	Collection date^a^	Voucher ID^b^	Total DNA (ng)	A_260_/A_280_	A_260_/A_230_	GenBank
*Lewinskya rupestris* c	2025/03/05	MAUAM 5865	6127 ± 966	1.97 ± 0.01	2.12 ± 0.08	PV520137–42
*Cardotiella secunda*	2022/06/04	MAUAM 5464	1440	1.94	2.04	PV520134
*Macrocoma tenuis*	2022/10/10	MAUAM 5881	2880	1.95	2.04	PV520143
*Macromitrium archeri*	2023/01/10	MAUAM 5694	1400	1.95	1.76	PV520144
*Nyholmiella obtusifolia*	2024/08/29	MAUAM 5700	4820	1.94	1.91	PV520145
*Orthotrichum anomalum*	2025/02/26	MAUAM 5875	9060	2.04	2.01	PV520146
*Pulvigera lyellii*	2025/03/09	MAUAM 5880	4240	1.98	2.02	PV520147
*Schlotheimia ferruginea*	2022/05/22	MAUAM 5879	2580	1.79	1.75	PV520149
*Ulota coarctata*	2024/08/10	MAUAM 5878	1750	1.90	1.89	PV520150
*Ulota hutchinsiae*	2024/08/06	MAUAM 5876	2720	1.92	1.83	PV520151
*Zygodon hookeri*	2023/01/12	MAUAM 5877	1460	1.93	1.94	PV520152
*Aulacomnium palustre*	2025/02/21	MAUAM 5883	3060	2.01	2.25	PV520132
*Bartramia pomiformis*	2025/03/09	MAUAM 5885	3960	2.03	2.13	PV520133
*Dendrocryphaea lamyana*	2024/09/25	MAUAM 5703	4800	1.97	2.25	PV520135
*Dicranum scoparium*	2025/02/21	MAUAM 5886	4680	2.01	2.22	PV520136
*Polytrichum juniperinum*	2025/03/08	MAUAM 5882	6540	1.95	1.98	PV520153
*Racomitrium aciculare*	2025/03/09	MAUAM 5884	1710	1.93	1.99	PV520148

^a^ The collection date is shown in the format year/month/day.

^b^ MAUAM = Universidad Autónoma de Madrid herbarium.

^c^ *Lewinskya rupestris* was tested with six replicates; average values are shown.

Additionally, we tested how the amount of starting material affects the effectiveness of the protocol. For this, we used our model species, *Lewinskya rupestris*, with four different sample amounts: 2, 8, 14, and 20 mg. Six replicates were included for each sample amount. A clear increase in DNA yield was observed in relation to the amount of starting material. Using 2, 8, 14, and 20 mg of sample, average total yields were 417 ± 70 ng, 3572 ± 1110 ng, 5787 ± 1028 ng, and 8290 ± 2097 ng, respectively. The mean A_260_/A_280_ ratios were 1.76 ± 0.05, 1.85 ± 0.02, 1.83 ± 0.02, and 1.89 ± 0.03, while the mean A_260_/A_230_ ratios were 1.47 ± 0.05, 1.99 ± 0.02, 2.01 ± 0.02, and 1.99 ± 0.09, respectively.

### DNA purity and quantity evaluation

The amount of DNA was measured using a Qubit 4 Fluorometer (Thermo Fisher Scientific, Waltham, Massachusetts, USA) using 1× dsDNA HS (High Sensitivity) Assay Kit (ng/L). Purity was measured with a NanoDrop ND‐1000 (Thermo Fisher Scientific) with absorbance ratios of A_260_/A_280_ and A_260_/A_230_. Typically, values between 1.8 and 2 are considered acceptable for the former, and above 2 for the latter (Desjardins and Conklin, 2010). To assess whether the differences obtained with the various modifications were statistically significant, descriptive statistics were obtained, and both parametric (ANOVA and Tukey's) and nonparametric (Kruskal–Wallis and Dunn's) tests were run.

We ran electrophoresis on an 0.8% agarose gel with 1× SYBR Safe DNA Gel Stain (Invitrogen, Waltham, Massachusetts, USA) and 1× Tritrack DNA Loading Dye (Thermo Fisher Scientific) to determine the success and quality of the DNA extraction. Absence of amplification inhibitors was assessed by Sanger sequencing of *rps4* (*rps5*, ATGTCCCGTTATCGAGGACCT [Nadot et al., 1994]; *trnaS*, TACCGAGGGTTCGAATC [Souza‐Chies et al., 1997]), a chloroplast locus that has been widely used for bryophyte phylogenetics and has been previously used in Orthotrichaceae (e.g., Draper et al., 2021; Aguado‐Ramsay et al., 2022). PCR amplifications were performed using Ready‐To‐Go PCR Beads (Amersham Pharmacia Biotech, Piscataway, New Jersey, USA) in a final volume of 25 µL, with 0.4 µM of primer and 1 µL of DNA. The PCR amplification protocol included 5 min at 94°C; 30 cycles of 30 s at 95°C, 1 min at 52°C, and 30 s at 68°C; and a last step of 7 min at 68°C. Amplification success was verified by electrophoresis, and PCR products were purified using Exol/FastAP (Thermo Fisher Scientific, Madrid, Spain) with 1 µL of exonuclease and 4 µL of FastAP enzymes per tube, applying 37°C and 85°C for 15 min each. Finally, forward reads were Sanger sequenced by Macrogen (Seoul, South Korea), a nucleotide BLAST search was run against the National Center for Biotechnology Information (NCBI) database, and the sequences were uploaded to GenBank (PV520132–PV520153; see Data Availability Statement).

## DISCUSSION

CTAB‐based methods are widely used to isolate DNA from plant tissues (Schenk et al., 2023). The Doyle and Doyle (1987) protocol was described as a minor modification from Saghai‐Maroof et al. (1984)—which was also a modification of a protocol by Murray and Thompson (1980)—with the only adjustments being the use of fresh leaf material and compensating water content to double the concentration of the extraction buffer. Associated publications are Doyle and Dickson (1987), the only version published in a conventional journal, and Doyle and Doyle (1990, 1991). Interestingly, similar protocols had been previously published by Taylor and Powell (1982) and Rogers and Bendich (1985). As stated by Doyle and Doyle (2019), it is only by a series of accidents that the two Doyle and Doyle (1987, 1990) protocols have been cited worldwide. Although they are widely used, CTAB extractions from some organisms still fail to produce pure high‐molecular‐weight DNA, which could be due to the unique chemical composition of secondary metabolites. Currently, the capacity to use HTS methodologies is frequently limited by the ability to extract unfragmented and high‐quality DNA (Kang et al., 2023; Pezzini et al., 2023); consequently, optimizing the CTAB protocol to remove secondary metabolites is essential (Jobes et al., 1995; Scott and Playford, 1996; Pirttilä et al., 2001; Horne et al., 2004; Dehestani and Kazemi Tabar, 2007; Japelaghi et al., 2011; Xin and Chen, 2012; Healey et al., 2014; Arruda et al., 2017; Inglis et al., 2018; Aboul‐Maaty and Oraby, 2019). We have developed a consensus protocol based on an extensive literature review while also evaluating various optimizations at different stages. The final protocol is detailed in Appendix 1. We discuss each step in detail below.

### Tissue preparation

The starting material is arguably the most crucial aspect of the protocol and should not be taken lightly. It is advisable to use young, unstressed tissue for optimal results. In our protocol, samples were cleaned with distilled water, placed in an Eppendorf tube, shaken in a Retsch Mixer Mill MM400, and air‐dried. Sample amounts vary significantly across published protocols; in our case, we initially used 20 mg. We also assessed the influence of the initial sample amount on DNA yield and purity, and found that even reduced quantities produced satisfactory results. For example, using 8 mg of our model species *Lewinskya rupestris*, we obtained a mean DNA yield of 3572 ng with 1.85 and 1.99 mean A_260_/A_280_ and A_260_/A_230_ ratios. The use of small sample sizes is desirable because it minimizes destructive impact on herbarium specimens (Rogers and Bendich, 1985) and reduces the risk of secondary metabolites or other interfering substances affecting sequencing (Jobes et al., 1995). For the grinding step, we tested several methods (results not shown), as this step is essential to ensure successful DNA isolation (e.g., Jones et al., 2023). We opted to use the automated grinder due to its capacity to process more samples in less time, minimizing contamination risk and assuring replicability. Grinding dry and frozen samples (−80°C or liquid nitrogen) resulted in a homogeneous fine powder, free of visible unground fragments. For further optimization, we experimented with varying quantities of 2‐mm and 3‐mm glass beads. Dry samples were ultimately flash‐frozen and ground in 2‐mL round‐bottom tubes containing 12 3‐mm glass beads using a Retsch Mixer Mill MM400 at 30 Hz, with two 2‐min cycles and tube orientation reversed between repetitions. The number of beads, grinding time, and number of repetitions can be increased for recalcitrant samples.

### Lysis

The main function of the lysis step is to disintegrate tissues, cells, and organelles to release their contents, particularly DNA. For the amount of applied buffer, we used 1 mL of buffer up to 100 mg of dry weight powder, following Murray and Thompson (1980). The incubation temperature and duration vary greatly among studies, but the most common combination is 60°C for 1 h (Schenk et al., 2023); however, Carey et al. (2023) determined that lower temperatures (55°C for 1 h) produced the best results. We tested several incubation times and found the differences to be relatively small, thus we concluded that incubation times can be adjusted according to the specific needs of the study, providing some flexibility to the protocol, although extending incubation might be useful for recalcitrant samples. Buffers usually include Tris, NaCl, EDTA, CTAB, and BME. While EDTA inactivates nucleases, preventing DNA degradation (Schenk et al., 2023), CTAB disrupts cell membranes and, when used with NaCl, also removes polysaccharides (Fang et al., 1992; Varma et al., 2007). We initially used the quantities proposed by Doyle and Doyle (1987), at 2% CTAB and 1.4 M NaCl, which are the most common concentrations. BME, which prevents protein oxidation and denatures proteins, is typically used at 0.2–0.5% (Schenk et al., 2023); however, we initially used 0.4%, as recommended by Larridon et al. (2020). Several other compounds have been added to the buffer in the modified CTAB protocols, especially PVP, an inert polymer that removes phenolic compounds (Loomis and Battaile, 1966). As indicated by Schenk et al. (2023), three different forms of this compound (PVP‐10, PVP‐40, and PVPP) are present in the literature. PVP‐40 is used most frequently, but the use of PVP‐10 has been recommended by some authors (e.g., Varma et al., 2007; Schenk et al., 2023). It has been suggested that PVPP may result in inferior DNA yield and purity (Schenk et al., 2023), although we have not found an article that has empirically evaluated them. Our results indicate that PVP‐10 may result in slightly higher DNA yields; most protocols use 1–2.5% (Schenk et al., 2023), and we initially used the highest value. The low A_260_/A_230_ value led us to evaluate whether higher concentrations of CTAB, BME, and PVP‐10 could be applied, as these might coprecipitate with DNA and either decrease final purity or inhibit PCR amplification (Demeke and Jenkins, 2010). Increasing the concentrations of CTAB and PVP‐10 did not have a significant impact on the results, probably due to the relatively high volume of buffer applied. Therefore, we only increased CTAB to 2.5%, as it showed slightly better outcomes and simplified the protocol by matching the PVP‐10 concentration. In regard to BME, higher concentrations appeared to reduce purity according to the A_260_/A_230_ ratio, and we therefore decided to maintain the initial concentration.

### Isolation

There are several ways to achieve DNA isolation, but the most common is chloroform–isoamyl alcohol (CIA) 24:1 (v/v), which should match the volume of the buffer (Schenk et al., 2023). The resulting aqueous phase contains the DNA, but also includes RNA, polysaccharides, and phenolic compounds, which can be problematic. We incorporate a second wash to facilitate RNA removal and minimize pipetting errors, with an RNase step between washes to break the RNA down into ribonucleosides, as its presence can interfere with primer sites during amplification (Jobes et al., 1995; Porebski et al., 1997). We include 2 μL of RNase (10 mg/mL, a final concentration of ~20 µg/mL) with an incubation of 30 min at 37°C, which is similar to what is recommended by Doyle and Doyle (1987) and most CTAB protocols (Schenk et al., 2023).

### Cleaning

This process can be carried out with commercial DNA cleaning kits, but it is often done with less expensive alcohols such as ethanol, which is preferred over isopropanol because solutes, such as sucrose and NaCl, can coprecipitate with the latter (Bult et al., 1992). The first step is to precipitate the DNA, for which we use isopropanol, as smaller quantities are needed (0.6–1 volume, compared to the 2–2.5 volumes of ethanol). For this step, we evaluated different precipitation times and temperatures. As already noted by other researchers (e.g., Shepherd and McLay, 2011; Kang et al., 2023), our results suggest that precipitating for longer times (i.e., several days) could be counterproductive, as it may also coprecipitate other compounds. On the other hand, modifying precipitation temperatures did not show a linear pattern in DNA yield or quality. In addition, we decided to evaluate the use of a salt to enhance precipitation in diluted DNA samples. There are several salts that can be applied; we selected sodium acetate at 0.3 M but did not observe any changes in the results. Most protocols incorporate two wash steps in 70–80% ethanol after precipitation (Schenk et al., 2023), which is critical to remove the CTAB that is soluble at around 80% (Rogers and Bendich, 1985). We ultimately decided to incorporate two washes at 70% and 95%, respectively, which facilitates pellet drying by minimizing water residues. We also evaluated the impact of an extra 70% ethanol wash, but this step did not show significant deviations.

### Elution

We recommend a final volume of nuclease‐free water or Tris‐EDTA (TE) buffer between 30 and 100 µL, depending on the expected DNA yield. Centrifugation with high force and longer time periods or overdrying the pellet could damage the DNA (Schenk et al., 2023). Some protocols heat the pellet in the elution buffer at 50–65°C for 5–120 min to resuspend the DNA, but Schenk et al. (2023) recommend lower temperatures to prevent DNA fragmentation. Several authors also point out that vortexing can degrade DNA (Khanuja et al., 1999; Sharma et al., 2000; Dehestani and Kazemi Tabar, 2007; Wang et al., 2011; Healey et al., 2014; Schenk et al., 2023), so we did not use it at any time during the protocol. On the other hand, CTAB carryover in the final extractant could increase the A_260_ absorbance readings, which will inflate the estimated concentration of DNA (Doyle and Doyle, 1990; Wilkie et al., 1993). Consequently, the Qubit system is preferred. In our particular case, NanoDrop quantification readings were generally around five times higher than those from the Qubit system.

### Additional tips

Our literature review revealed that there is insufficient information to allow proper replicability of most protocols. We found that the initial sample treatment is perhaps the most important phase of the protocol. Fine‐tuning the grinding techniques according to the capabilities of each laboratory is also vital. In any case, throughout all the extractions carried out in this study, we observed a variability between runs of DNA extractions that cannot be explained, despite efforts to keep all conditions as consistent as possible (e.g., same operator, same sample quantity). This is likely attributable to several factors, including the quality of the starting material (which could be influenced by the collection conditions), the effectiveness of tissue disruption during the grinding step, and minor deviations in the protocol—such as pipetting inaccuracies or incomplete buffer dissolution—which can accumulate and impact the final outcome. This limits the comparability of results between DNA extraction runs, a detail that is often omitted in studies comparing DNA extraction protocols, which rarely specify whether results come from a single run or multiple runs. To ensure correct comparability, it is important to make sure that the starting material is as similar as possible and that all other conditions remain the same, if possible, in order to study the modifications in the same DNA extraction run.

## CONCLUSIONS

We present an optimized, rapid, and efficient DNA extraction protocol adapted for 2‐mL tubes and 24‐sample batches, while remaining flexible for different laboratory settings. The protocol yields high‐quality DNA suitable for high‐throughput sequencing and other molecular analyses. It was developed through systematic testing of individual steps, enabling both improvement and simplification. Key findings from this optimization phase include: the incubation times can be adjusted to accommodate daily laboratory workflows, with one hour proving sufficient for optimal results; PVP‐10 was identified as the most effective polyvinylpyrrolidone variant; and the concentrations of PVP‐10, CTAB, and BME were evaluated for optimal performance. Extended precipitation times were found to decrease DNA purity, while precipitation temperature and the addition of salt had no impact. Although the protocol was specifically designed for the family Orthotrichaceae, it has been preliminarily tested on representatives of other families and is likely to perform well with a broad range of bryophyte taxa. Therefore, this method potentially represents a valuable tool for molecular studies involving recalcitrant samples.

## AUTHOR CONTRIBUTIONS

P.A.‐R., F.L., and I.D. designed the study. P.A.‐R. and I.D. gathered the necessary equipment for the laboratory. P.A.‐R. and F.L. collected the extracted samples. P.A.‐R. and M.C. performed DNA isolation, M.C. and I.D. performed amplification and sequencing, and P.A.‐R. analyzed the data and wrote the initial draft of the manuscript. All authors reviewed and approved the final version of the manuscript.

## Supporting information


**Appendix S1.** Information associated with the samples used in the study.
**Appendix S2.** Descriptive statistics and both parametric and nonparametric tests for the statistically significant results from the protocol comparisons.
**Appendix S3.** Descriptive statistics and both parametric and nonparametric tests for the statistically significant results from the protocol optimizations.

**Appendix S4.** Results of the protocol optimization phase.

## Data Availability

Sequences obtained in this study were submitted to GenBank (PV520132–PV520153).

## Appendix 1 Step‐by‐step version of the final protocol.

**Reagents needed:**

**CTAB extraction buffer** (see Table A1): 20 mM EDTA (pH 8.0, molecular weight [MW]: 372.24), 100 mM Tris‐Cl (pH 8.0), 1.4 M NaCl (MW: 58.44), 2.5% cetyltrimethylammonium bromide (CTAB) (w/v), 0.4% β‐mercaptoethanol (BME) (v/v), and 2.5% polyvinylpyrrolidone (PVP) (w/v, MW: 10,000)

**Table A1:** Quantities needed to mix the CTAB extraction buffer.

Solution/Reagent	Stock	1 sample	In µL	24 samples	Final concentration
CTAB		0.025 g		0.6 g	2.5%
PVP‐10		0.025 g		0.6 g	2.5%
NaCl	5 M (292.2 g/L)	0.28 mL	280 µL	6720 µL	1.4 M
EDTA (pH 8.0)	0.5 M (186.2 g/L)	0.04 mL	40 µL	960 µL	20 mM
Tris‐Cl (pH 8.0)	1 M	0.1 mL	100 µL	2400 µL	100 mM
BME		0.004 mL	4 µL	96 µL	0.4%
H_2_O		0.576 mL	576 µL	13824 µL	
Total volume		1 mL	1000 µL	24 mL	

**Tris‐EDTA buffer**: 1 mM EDTA, 10 mM Tris‐Cl

**Other reagents**: Chloroform:isoamyl alcohol (CIA) (24:1 v/v), isopropanol, ethanol (70% and 95%), RNase (10 mg/mL). We maintain a stock of NaCl at 5 M (292.2 g/L), EDTA at 0.5 M (186.2 g/L), and Tris‐Cl at 1 M.

**Equipment needed:**

- Round‐bottom Eppendorf tubes (2 mL), screw‐cap microcentrifuge tubes (1.6 mL), and conical centrifuge tubes (50 mL)
- Bead mill homogenizer (alternatively, a mortar and pestle may be used) (e.g., Retsch Mixer Mill MM400; Retsch, Haan, Germany)
- Dry block heater
- Liquid nitrogen or a −80°C freezer
- Microvolume spectrophotometer (e.g., NanoDrop ND‐1000; Thermo Fisher Scientific, Waltham, Massachusetts, USA)
- Qubit 4 Fluorometer (Thermo Fisher Scientific) with 1× dsDNA HS (High Sensitivity) Assay Kit (ng/L)
- Refrigerated centrifuge

**Precautions required for this activity:** Personal protective equipment is required throughout the procedure. Nitrile gloves should be worn at all times. Eye protection is recommended at all times. Any bottle or tube containing BME or chloroform must be opened in a chemical fume hood.

**Waste disposal:** All solvents used in the procedure must be discarded in properly labeled hazardous waste containers, and all solid items such as pipette tips, gloves, and paper towels that have come into contact with these chemicals must be disposed of in the solid hazardous waste container. No contaminated materials should be left out once a step is complete; all waste must be cleared promptly and correctly.

As the protocol developed by the McDaniel Lab indicates (https://mcdaniellab.biology.ufl.edu/data/), “A DNA extraction is only as good as the least successful step. Good extractions are the result of each step being done carefully.” Good luck! [Correction added on 19 December 2025, after first online publication: The text was amended to properly acknowledge the source of the quoted text.]

**Preparation steps:**

1. Prepare the CTAB buffer (Table A1). Let the solution incubate in an oven at 50°C for at least 1 h (ideally, leave overnight at 37°C).
2. Verify that there are sufficient volumes of isopropanol and 70% and 95% ethanol in the freezer and CIA (24:1) stored in proper container. Place a rack inside the freezer. Turn on the heat block and set it to 55°C.
3. Add BME to the buffer just before use.
4. Grind the samples using a bead mill homogenizer in 2‐mL round‐bottom tubes.
  **Extraction procedure:**
5. Add 900 µL of CTAB extraction buffer and incubate at 55°C for at least 1 h, inverting the tube occasionally. Longer incubation times might result in higher DNA yields, so consider overnight incubation for recalcitrant samples.
6. Briefly centrifuge the samples to ensure all contents settle at the bottom of the tube; this prevents material from remaining on the lid and thereby minimizes the risk of cross‐contamination.
7. Add 800 µL of CIA (24:1 v/v) and mix thoroughly by inversion.
8. Centrifuge at 14,000 rpm for 10 min. Label new tubes so they are ready for the next steps.
9. Transfer the aqueous supernatant to a new Eppendorf tube. Add 2 μL of RNase (10 mg/mL) and incubate at 37°C for 30 min.
10. Repeat the chloroform wash with 700 μL (equal volume) of CIA.
11. Transfer the aqueous supernatant to a new 1.6‐mL screw‐cap microcentrifuge tube. Add 600 µL (0.6−1 volumes) of ice‐cold isopropanol and place the tubes in a prechilled rack. Invert the tubes and incubate in a freezer or refrigerator for at least 2 h. Longer precipitation times can be applied, but minimizing it is highly recommended, as other compounds can coprecipitate.
12. Centrifuge for 10 min at 12,000 rpm. If there is sufficient DNA, a visible pellet should have formed at the bottom of the tube. The absence of a visible pellet does not necessarily mean the extraction has failed, but it does indicate that the DNA yield is likely to be low.
13. Pour off the isopropanol, being careful not to dislodge the pellet, and add 500 µL of ice‐cold 70% ethanol.
14. Centrifuge for 5 min at 12,000 rpm. Pour off the ethanol, being careful not to dislodge the pellet. Repeating the 70% ethanol wash is recommended if the DNA pellet is large and still green.
15. Repeat the ethanol wash with 500 µL of 95% ethanol. If no visible pellet was formed, this second wash can be skipped. Leave the pellet to dry for 30–60 min, and then add 30–100 µL of preheated Tris‐EDTA (TE) buffer or nuclease‐free water (depending on the expected DNA yield and time of storage). Samples can be placed in a heating block at 40–50°C to help the pellet dissolve. The required time may vary depending on the conditions, but 15 min is usually sufficient if gentle mixing is used to facilitate the process.
16. Run a gel and use a NanoDrop and Qubit 4 Fluorometer to assess DNA purity and concentration, as described in the Methods section.
