# Supplementary figures and images for: Optimizing DNA extraction protocols for bryophytes: Insights from Orthotrichaceae
Source: Appl Plant Sci. 2025 Nov 4;13(6):e70028. doi: 10.1002/aps3.70028 (PMC12747542; doi:10.1002/aps3.70028)

**Appendix S4.** Results of the protocol optimization phase.


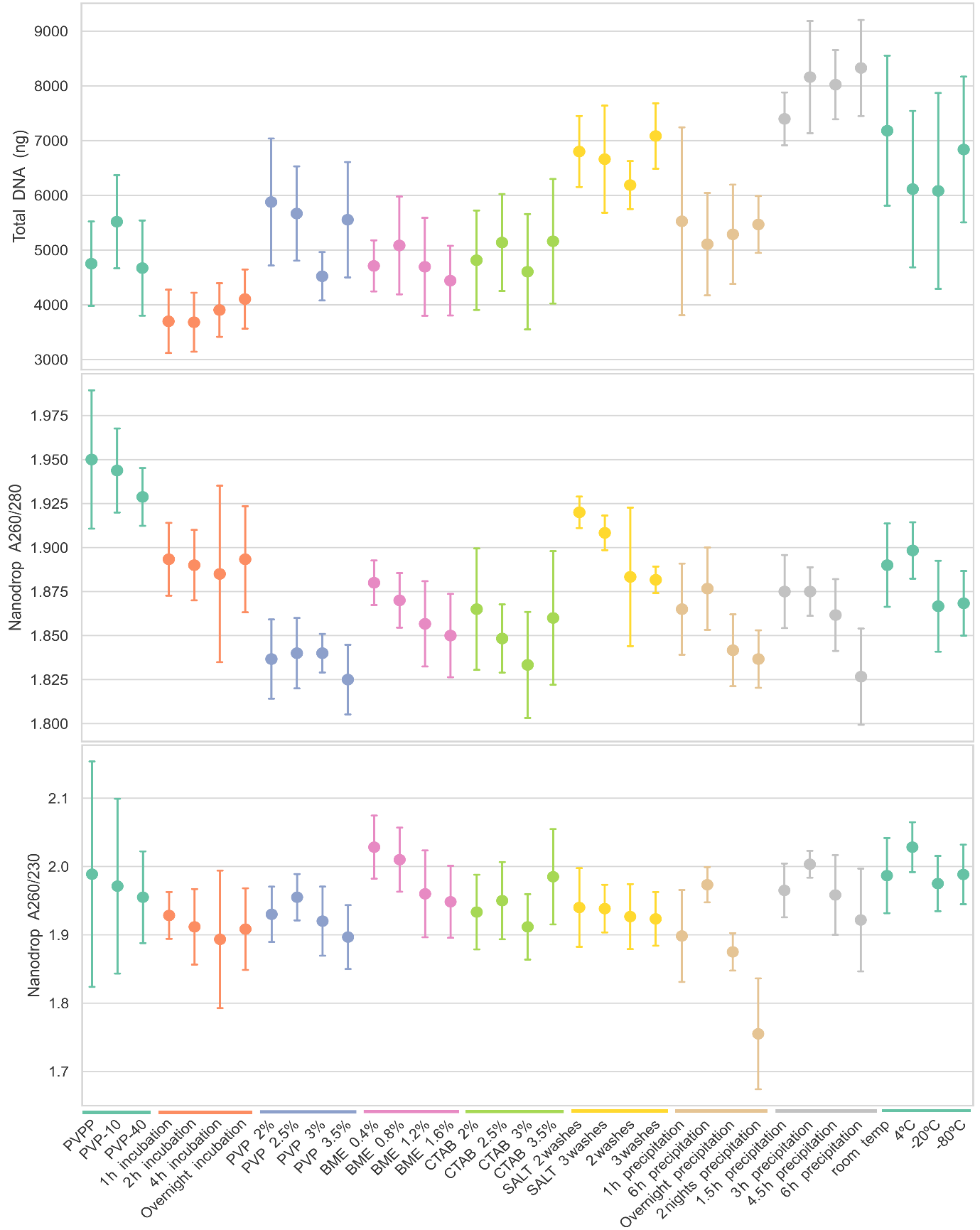

Supplement: Supplementary file 2 — Appendix S4. Results of the protocol optimization phase. [file APS3-13-e70028-s002.docx]
